# Alkaline Pre-Impregnation and Inter-Stage Washing for Enhanced Soda-AQ Pulping of Oil Palm EFB

**DOI:** 10.1021/acsomega.6c05915

**Published:** 2026-09-04

**Authors:** Yun Jie Chuah, Shi Teng Ng, Yitong Niu, Xiang Yi Liew, Cheu Peng Leh

**Affiliations:** Division of Bioresource Technology, School of Industrial Technology, 26689 Universiti Sains Malaysia, Minden, Penang 11800, Malaysia

## Abstract

Alkaline pre-impregnation prior to soda-anthraquinone pulping of oil palm empty fruit bunches enables conversion into highly delignified, high-strength cellulosic matrices. A 2^3^ factorial design was established to evaluate the main and interaction effects of pre-impregnation variables—temperature, time, and alkali charge—against conventional soda-anthraquinone pulping. While pulp yield was invariant, pulp and paper properties were strongly governed by distinct main and two-way interaction effects. Crucially, temperature dominated the pulp viscosity, burst, and tearing indices. Leveraging this insight, an inter-stage washing step was integrated while fixing temperature at a lower level to preserve fiber integrity, with the remaining conditions anchored by the factorial model. This integrated sequence significantly outperformed conventional and unwashed two-stage alternatives by removing dissolved lignin, while maximizing the retention of structural hemicelluloses to promote fiber bonding, yielding a lower kappa number (10.4) alongside elevated tensile (22.68 N·m/g), burst (4.40 kPa·m^2^/g), and tearing (8.36 mN·m^2^/g) indices. These findings provide an efficient, low-temperature framework for agricultural residue valorization into high-strength bio-based materials.

## Graphical abstract

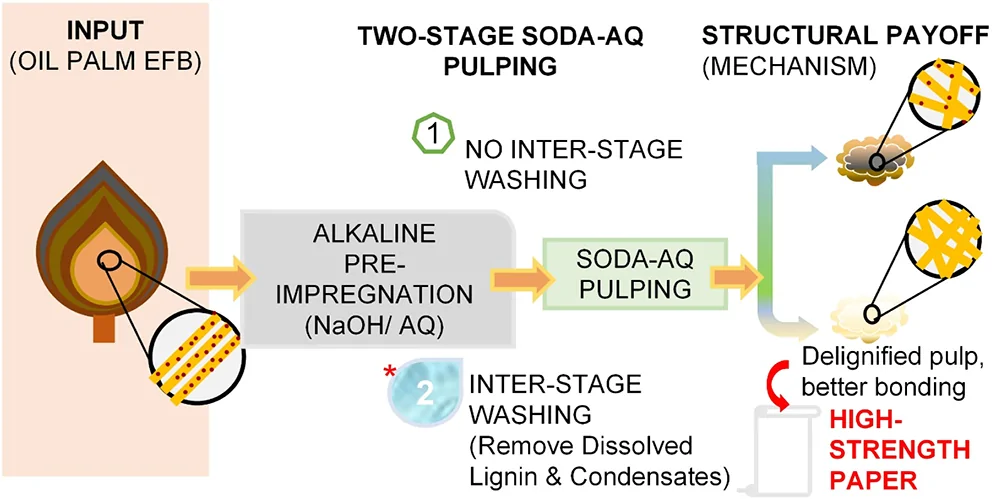

## 1 Introduction

According to Statista Research Department,^1^ the global production of paper-based commodities, such as tissue, packaging paper, and paperboard, has grown from about 60 million metric tons in 1961 to nearly 200 million metric tons in 2022. Wood pulp remains the predominant raw material for worldwide paper production, with a market value exceeding 160 billion U.S. dollars.^1^ Both softwood and hardwood, therefore, play a critical role in sustaining the paper industry. However, the heavy dependence on wood resources poses a substantial environmental challenge, as extensive logging leads to deforestation and associated ecological impacts. To mitigate these concerns, sustainable forest management policies have been encouraged and implemented globally, including mandatory reforestation programs and forest certification schemes, for instance Forest Stewardship Council (FSC), the Programme for Endorsement of Forest Certification (PEFC). Despite these efforts, the combined forest area certified under PEFC^2^ and FSC,^3^ totals approximately 467 million hectares, representing only about 11% of the global forest area of 4.14 billion hectares,^4^ indicating that the coverage of certified sustainable forest management remains relatively limited. Consequently, the utilization of non-wood biomass, particularly from agricultural residues such as sugarcane bagasse, rice straw, oil palm (*Elaeis guineensis*) empty fruit bunches (EFB) and trunks, wheat and cotton stalks can offer a viable pathway to reduce reliance on wood resources.^5^

According to Statista,^6^ Malaysia is the second-largest producer of crude palm oil (CPO) after Indonesia, with production approaching 19 million tonnes annually. Among the biomass residues generated, EFB represent one of the major agro-industrial lignocellulosic by-products of the palm oil industry, with approximately 7.3 million tonnes produced in 2023.^7^ Although oil palm fronds (59.6 million tonnes) and trunks (10.5 million tonnes) are generated in larger quantities, EFB offers a logistical advantage because it is produced directly at milling facilities during oil extraction, eliminating the need for additional collection and transportation from plantations.

EFB is characterized by relatively high holocellulose content (82–84%) and lower lignin content (15–18%) compared with wood materials,^8^ making it a promising feedstock for pulp and paper production,^9^ as well as for bioenergy and biochar applications.^10,11^ Despite its potential, reports from the Ministry of Plantation and Commodities and Malaysian Palm Oil Board (MPOB)^7,12^ indicate that EFB remains underutilized, with approximately 41.2% returned to plantations for mulching and only 3.8% incinerated, resulting in utilization rates below 50%.^7^ Therefore, valorization of EFB as a pulp and paper raw material represents a sustainable and economically attractive alternative compared to wood resources, capitalizing on its favorable chemical composition and inherent logistical advantages.

Since the late 20th century, the expansion of the oil palm industry has indirectly stimulated research on utilization of its biomass by-products for the production of various fiber- or cellulose-based products, including pulp and paper.^10,11^ Extensive studies have evaluated the feasibility of producing paper from EFB employing different chemical pulping methods, such as kraft, soda, soda-anthraquinone (soda-AQ), sulfite, as well as organosolv pulping.^13–16^ The production of hybrid or mechanical pulps from EFB has also been reported.^17,18^ Although EFB fibers (0.67 mm) are considerably shorter than conventional hardwood (0.83 mm) and softwood (2.39 mm) benchmarks, their favorable anatomical properties—namely a wide lumen cavity relative to fiber width and a low Runkel ratio of 0.59—align perfectly with the structural baselines established by the MPOB.^19^ Thus, these morphological features make the fibers highly flexible and easily collapsible, which is advantageous for forming strong fiber-to-fiber bonds. Nevertheless, its short fiber length remains a limiting factor for achieving high paper strength.

In our previous works, we demonstrated that alkaline pre-impregnation prior to soda-AQ pulping—an alternative or modified pulping method—significantly enhanced pulping efficiency and improved the paper strength properties of pulp produced from kenaf.^20^ This modified pulping approach facilitates delignification while minimizing the degradation of cellulose and hemicellulose. Various pre-impregnation techniques, including steam explosion, acid hydrolysis, alkaline treatment and organosolv processes, have been widely investigated.^21^ However, to date, the alkaline pre-impregnation before the pulping efficiency of EFB has not been systematically reported.

This work aims to investigate the potential of a two-stage soda-AQ pulping process for improving the pulping performance of EFB. The study systematically evaluates the impact of an alkaline pre-impregnation stage on pulp and paper properties in comparison to conventional one-stage soda-AQ pulping. To screen the independent variables and analyze the main and interaction effects of temperature, time, and alkali charge. Furthermore, this research examines the role of an inter-stage washing step following alkaline pre-impregnation to determine its influence on delignification efficiency, pulp yield, and resulting pulp and paper strength properties. By comparing these configurations, this study seeks to establish an efficient pretreatment strategy for enhancing the valorization of EFB in papermaking.

## 2 Experimental Section

### 2.1 Raw Materials

EFB in loose fibrous stand form was supplied by United Palm Oil Industries Sdn. Bhd. located in Penang, Malaysia. The EFB strand was first soaked overnight in tap water at a liquor-to-material (L:M) ratio of 1:15 and then washed to eliminate dirt and parts of water-soluble extractives, followed by air drying at room temperature for 3 to 4 days. The washed and dried EFB was then kept in plastic bags before use. The moisture content of the EFB was determined according to TAPPI T 264.

### 2.2 Pulp

The pre-impregnation soda-AQ pulping with L:M of 7:1 was carried out using sodium hydroxide (NaOH) as the sole cooking chemical in a 4-liter stainless steel static digester (NAC Autoclave Co., Ltd., Japan). The total alkali charge was fixed at 25% (based on the oven-dry weight of biomass), where the alkali charge in the subsequent soda-AQ pulping was adjusted to account for the amount applied during the pre-impregnation stage. The experimental design for alkaline pre-impregnation is summarized in Table 1, which shows a two-level factorial design with seven experimental conditions for three parameters: impregnation temperature, *T* (50.0 and 65.0 °C), impregnation time, *t* (2.0 and 4.0 h), and alkali charge, *A*_*c*_ (5.0 and 8.0%). The subsequent soda-AQ pulping was conducted under fixed conditions: 160 °C, 2 h, L:M of 8:1, and 0.1% anthraquinone.

**Table 1:** Experimental Conditions of Alkaline Pre-Impregnation—Soda-AQ Pulping without Inter-Stage Washing of EFB with Both Coded and Actual Variable Levels and Alkali Charge Topped up for Soda-AQ Pulping

Variable code			Alkaline pre-impregnation			Soda-AQ pulping
*T*	t	*A*_*c*_	Temperature, °C	Time, h	Alkali charge, %	Alkali charge (topped up), %
1	1	1	50.0	2.0	5.0	20.0
1	1	–1	50.0	2.0	8.0	17.0
1	–1	1	50.0	4.0	5.0	20.0
1	–1	–1	50.0	4.0	8.0	17.0
–1	1	1	65.0	2.0	5.0	20.0
–1	1	–1	65.0	2.0	8.0	17.0
–1	–1	1	65.0	4.0	5.0	20.0
–1	–1	–1	65.0	4.0	8.0	17.0

Upon completion of pulping, the pressure was released, and the temperature was rapidly reduced to below 100 °C. The brown stock pulp was diluted with approximately 3 L of tap water, transferred to a laboratory hydro-pulper, and washed for 10 min to remove degraded lignin and spent liquor. The pulp slurry was further rinsed with tap water on a stainless-steel 200-mesh filter until no soapy feel remained. The slurry was then disintegrated for 2000 revolutions according to TAPPI T205 and screened using a flat-plate Somerville screen with 0.15 mm slits in accordance with TAPPI T 275. Both the accepts (pulp yield) and rejects (reject yield) were collected. After spin-drying, the moisture content of the pulps was determined as described previously in Section 2.1 (according to TAPPI T264) to calculate the oven-dried weight of screened accepts/rejects and pulp yield obtained according to eqs 1 to 2.

1 Moisture content(%)=[(AD Weight
of Screened Accepts(g)−OD Weight of Screened
Accepts(g))/AD Weight of Screened Accepts(g)]×100%

2 $$Pulp\;yield\;(\%)=\frac{OD\;Weight\;of\;Screened\;Accepts\;\;(g)}{OD\;Weight\;of\;EFB\;(g)}\times 100\%$$

### 2.3 Alkaline Pre-Impregnation

#### 2.3.1 Without Inter-Stage Washing

As listed in Table 1, the EFB fibrous strand was soaked in a selected alkali charge, temperature, and time. The L:M applied was 7:1. After alkaline pre-impregnation, a diluted NaOH solution was directly topped up to achieve the desired total alkali charge of 25% at a L:M of 8:1 without washing, and the soda-AQ pulping (Section 2.2) was then continued under a fixed condition.

#### 2.3.2 With Inter-Stage Washing

The same alkaline pre-impregnation conditions and procedure described in Section 2.3.1 were carried out until completion. After the end of the impregnation stage, the biomass was thoroughly washed with tap water to remove all spent liquor or residual alkali. The washed biomass was then spin-dried, and its moisture content was determined. Soda-AQ pulping (Section 2.2) was subsequently performed by readjusting the alkali charge and L:M to meet the required condition.

### 2.4 Pulp Properties

The kappa number was determined according to TAPPI T236. The carbohydrate composition was analyzed using TAPPI T249. The pulp viscosity was measured following TAPPI T230, and the pulp freeness was determined using TAPPI T227.

### 2.5 Handsheet Making and Testing

Ten handsheets were prepared for each sample following TAPPI T205, air-dried and conditioned according to TAPPI 402 at 23 ± 1 °C and relative humidity of 50 ± 2% for at least 3 h. Five to seven well-formed handsheets per sample were selected as replicates to proceed with testing accordance with TAPPI T220. Grammage of handsheets was conducted based on TAPPI T410. The thickness of handsheets was conducted based on TAPPI T411. Density was calculated by dividing the grammage by the handsheet thickness. Both TAPPI opacity and printing opacity of handsheets were determined according to TAPPI T425. Brightness was measured using TAPPI T452. Mechanical strength properties evaluated were tensile strength (TAPPI T494), tear strength (TAPPI T414), and burst strength (TAPPI T403). To minimize the effect of variations in handsheet grammage on strength properties, the strength indices were calculated by dividing the respective strength values by the grammage, as shown by eqs 3–5

3 $$Tensile\;index\left(\frac{Nm}{g}\right)=\frac{Tensile\;Strength\;(\frac{N}{m})}{Grammage\;(\frac{g}{m^{2}})}$$

4 $$Burst\;index\;(\mathrm{kPa}\cdot \frac{m^{2}}{g})=\frac{Burst\;Strength\;(kPa)}{Grammage\;(\frac{g}{m^{2}})}$$

5 $$Tearing\;index\left(mN\cdot \frac{m^{2}}{g}\right)=\frac{Tear\;strength\;(mN)}{Grammage\;(\frac{g}{m^{2}})}$$

### 2.6 Experiment Design

A full two-level factorial design (2^3^) was used to statistically analyze the effect of alkaline pre-impregnation variable with the aid of computer software, Design Expert (Stat-Ease, Inc., USA). The three independent variables were impregnation temperature (*T*), impregnation time (*t*), and alkali charge (*A*_*c*_). The seven response variables examined were pulp yield, kappa number, pulp viscosity, tensile index, burst index, tearing index, and brightness. Table 1 presents the seven experimental conditions calculated by the software. The coded values of the independent variables were derived from eqs 6–8.

6 $$T_{\mathrm{code}}=\frac{\mathrm{Temp.}-57.5^{{}^{\circ}}C}{7.5{}^{\circ}C}$$

7 $$t_{\mathrm{code}}=\frac{\mathrm{Time}-3.0\;h}{1.0\;h}$$

8 $$A_{c}\mathrm{code}=\frac{\mathrm{Alkali Charge}-6.5\%}{1.5\%}$$

## 3 Results and Discussion

### 3.1 Two-Level Factorial Design Analysis

The experimental design and corresponding response data for the unwashed two-stage soda-AQ sequence are compiled and presented in Table 2. By adopting this experimental design, the significant influence of each parameter and their interactions on the properties of the produced pulps could be identified.

**Table 2:** Experimental Design Matrix in Coded and Actual Levels for Alkaline Pre-Impregnation of EFB—Soda-AQ Pulping without Inter-Stage Washing and the Resulting Pulp and Handsheet Properties

Variable code			Variable			Pulp properties			Handsheet properties			
*T*	*t*	*A*_*C*_	Temperature, °C	Time, h	Alkali charge, %Alkali Charge, %	Pulp yield, %	Kappa number	Pulp viscosity, cP	Tensile index, Nm/g	Burst index, kPa·m^2^/g	Tearing index, mN m^2^/g	Brightness, %
Control			Control			50.0	13.9	26.16	16.89	3.24	5.95	42.43
1	1	1	50.0	2.0	5.0	52.5	12.9	23.67	19.79	3.52	5.95	43.91
1	1	–1	50.0	2.0	8.0	52.5	12.4	21.44	18.24	3.62	6.20	46.16
1	–1	1	50.0	4.0	5.0	51.9	12.5	25.05	18.64	3.55	6.87	44.20
1	–1	–1	50.0	4.0	8.0	53.8	13.0	23.42	15.25	3.46	6.90	43.59
–1	1	1	65.0	2.0	5.0	50.9	13.4	24.54	17.44	3.77	6.87	44.20
–1	1	–1	65.0	2.0	8.0	54.0	12.9	24.84	19.08	3.83	6.94	45.73
–1	–1	1	65.0	4.0	5.0	52.6	12.4	25.10	19.64	3.80	6.96	43.70
–1	–1	–1	65.0	4.0	8.0	52.6	12.8	25.24	19.17	3.68	7.36	44.32

Factorial models were developed for seven response variables, which were three pulp properties (pulp yield, kappa number and pulp viscosity) and four paper properties (tensile index, burst index, tearing index and brightness), using Design-Expert software (Stat-Ease, Inc. USA). The significant individual and interaction effects on each response variable were identified using half-normal probability plots. Variables with large absolute effects deviating from the linear trend were considered significant, whereas variables clustered near zero were classified as insignificant. By selecting these outlying variables, the responses were fitted using a linear model with two-factor interactions (2FI), as described by the following general regression equation template:

9 $$Y=\beta_{0}+\beta_{1}T+\beta_{2}t+\beta_{3}A_{C}+\beta_{12}Tt+\beta_{13}TA_{C}+\beta_{23}tA_{C}$$

where *Y* is the predicted response; β_0_ is the intercept; β_1_, β_2_, and β_3_ are the linear coefficients for temperature (T), time (t), and alkali charge (A_c_), respectively; and β_12_ β_13_, and β_23_ represent the multi-variable interaction coefficients.

Based on the coefficient estimates determined via analysis of variance (ANOVA), the final empirical predictive regression models for each pulp and paper property in terms of coded factors are established as follows in eqs 10–16:

10 $$Pulp\;yield\;(\%)=+52.64+0.5537T+0.1687t-0.0950A_{c}-0.2512Tt+0.1500TA_{c}-0.0600tA_{c}$$

11 $$kappa\;number=+12.79+0.0875T-0.1100t-0.0125A_{c}-0.1625Tt+0.2375tA_{c}$$

12 $$Pulp\;\mathrm{viscosity}\;(cP)=+24.16+0.7675T+0.5400t-0.4275A_{c}-0.3000Tt+0.5375TA_{c}$$

13 $$Tensile\;index\;(Nm/g)=+18.41+0.4262T-0.2313t-0.4713A_{c}+0.8038Tt+0.7638TA_{c}-0.4937tA_{c}$$

14 $$Burst\;index\;(kPa\cdot m^{2}/g)=+3.65+0.1162T-0.0313t-0.0063A_{c}-0.0087TA_{c}-0.0462tA_{c}$$

15 $$Tearing\;index\;(mN\cdot m^{2}/g)=+6.76+0.2762T+0.662t+0.0938A_{c}-0.1387Tt$$

16 $$Brightness\;(\%)=+44.48-0.5238t+0.4737A_{c}-0.4712tA_{c}$$

Table 3 summarizes the ANOVA of the reduced factorial models developed for the seven response variables, together with their corresponding coefficient (CE) for each significant variable. All established models incorporated both individual (main effect) and combined variables (interaction effects). Except for the pulp yield model, the ANOVA verified that, the reduced models built for the other six factorial models listed as eqs 11–16 (kappa number, pulp viscosity, tensile index, burst index, tearing index and brightness) were statistically significant, with probability values (prob. >F) below 0.05. Because the experimental matrix is an unreplicated design without center points, a formal lack-of-fit test is not applicable, while with acceptable coefficients of determination (R^2^) ranging from 0.9164 to 0.9998, close agreement between Adjusted and Predicted R^2^ with difference less than 0.2, and Adequate Precision ratios above 4.0, indicating strong predictive capability and valid diagnostic checks and adequate model fit (Table 3).

**Table 3:** Statistical Assessment and Analysis of the Variable to Response (Reduced Model)^a^ ^b^

	Response													
	Pulp yield, %		Kappa number		Pulp viscosity, cP		Tensile index, Nm/g		Burst index, kPa·m^2^/g		Tearing index, mN m^2^/g		Brightness, %	
Variable	CE	Prob. > F	CE	Prob. > F	CE	Prob. > F	CE	Prob. > F	CE	Prob. > F	CE	Prob. > F	CE	Prob. > F
Intercept	52.64	-	12.79	-	24.16	-	18.41	-	3.65	-	6.76	-	44.48	-
*T*-Temperature	0.5537	0.5759	0.0875	0.0198	0.7675	0.0101	0.4262	0.0503	0.1162	0.0001	0.2762	0.0075	-	-
*t*-time	0.1687	0.8875	–0.1100	0.0121	0.5400	0.0200	–0.2313	0.0923	–0.0313	0.0016	0.2662	0.0083	–0.5238	0.0150
*A*_*c*_-Alkali Charge	–0.0950	0.9147	–0.0125	0.4226	–0.4275	0.0314	–0.4713	0.0455	–0.0063	0.0377	0.0938	0.1159	0.4737	0.0209
*Tt*	–0.2512	0.8346	–0.1625	0.0059	–0.3000	0.0609	0.8038	0.0267	-	-	–0.1387	0.0476	-	-
*TA*_*c*_	0.1500	0.8512	-	-	0.5375	0.0202	0.7638	0.0281	–0.0087	0.0198	-	-	-	-
*tA*_*c*_	–0.0600	0.9596	0.2375	0.0028	-	-	–0.4937	0.0434	–0.0462	0.0007	-	-	–0.4712	0.0213
Model Prob. > F	0.9332		0.0075		0.0206		0.0455		0.0005		0.0013		0.0127	
R^2^	0.5457		0.9970		0.9917		0.9994		0.9998		0.9697		0.9164	
Adjusted R^2^	–2.1798		0.9894		0.9710		0.9959		0.9993		0.9293		0.8537	
Predicted R^2^	–28.0721		0.9517		0.8674		0.9623		0.9970		0.7844		0.6657	
Adjusted R^2^ - Predicted R^2^	-		0.0377		0.1036		0.0336		0.0023		0.1449		0.1880	
Adequate Precision	1.1500		33.4800		19.4100		50.8400		120.8400		13.3200		7.7800	

a CE-Coefficient estimation.

b Prob. > F-p-value.

### 3.2 Effects of Pre-Impregnation Variables on Pulp and Paper Properties

#### 3.2.1 Pulp Yield

The pulp yield obtained from the alkaline pre-impregnation – soda-AQ pulping without inter-stage washing (two-stage soda-AQ pulping) ranged from 50.9 to 54.0% (Table 2). The ANOVA indicated that pulp yield was not significantly influenced by the selected pre-impregnation variables within the studied ranges (p-value > 0.05), with a negative Predicted R^2^ and a low Adequate Precision (1.15), suggesting that the variation in yield within the tested design space was dominated by experimental noise or requires a higher-order (RSM) design to be accurately mapped. Consequently, a factorial model for pulp yield could not be established (Table 3).

Although the effects of individual variables were statistically insignificant, the two-stage soda-AQ pulping process resulted in slightly higher pulp yield compared to conventional single-stage soda-AQ pulping (one-stage soda-AQ pulping) (50.0%). The observed yield retention can be attributed to the mild alkaline pre-impregnation conditions applied —low temperature (50.0–65.0 °C), moderate alkali charge (5.0–8.0%), and extended impregnation time (2.0–4.0 h) prior to the pulping. These conditions promote effective alkali penetration into the fiber structure while limiting carbohydrate, especially hemicellulose, degradation. As verified in Table 5, the hemicellulose content increased from 24.9% to 26.1% when the pulp is pre-impregnated with alkali. In addition, as stated by Ang et al.,^20^ partial alkali consumption during the pre-impregnation stage can reduce the effective alkali concentration during the subsequent soda-AQ pulping stage conducted at elevated temperatures, thereby minimizing excessive dissolution of cellulose and hemicellulose, followed by an increased pulp yield.

#### 3.2.2 Kappa Number

The factorial model (eq 11, Table 3) shows that impregnation time (*t*) and alkali charge (*A*_*c*_) exerted a negative effect on the kappa number, indicating enhanced delignification, whereas the impregnation temperature (*T*) was positive effect. The increase in kappa number with increasing temperature may be attributed to the Arrhenius effect during the first stages of impregnation, whereby elevated temperatures accelerate alkali-consuming reactions. These reactions may include the neutralization of organic acids derived from hemicelluloses, deacetylation and saponification reactions, cellulose peeling, as well as reactions with extractives and readily accessible lignin structures, thereby reducing the effective residual alkali available for bulk delignification during the subsequent soda-AQ pulping stage.^22^ Among these variables, *A*_*c*_ exhibited the weakest influence, with a non-significant p-value of 0.4226. In addition, two interaction terms, namely time-alkali charge (*tA*_*c*_) and temperature–time (*Tt*), were found to be statistically significant, with p = 0.0028 and 0.0059, respectively.

The interactive effects of *tA*_*c*_ and *Tt* on the kappa number are illustrated in Figure 1a and Figure 1b. Although increasing *t* and *A*_*c*_ individually promoted delignification, their combined effects exhibited a positive coefficient (+0.2375), indicating a reduction in delignification efficiency when both variables were at high levels. Figure 1a demonstrated that the kappa number reduction was more pronounced when one variable was at a high level while the other remained low. These results revealed that effective delignification during subsequent soda-AQ pulping requires sufficient chemical penetration during pre-impregnation while maintaining adequate effective alkali for the high-temperature pulping stage. Such conditions could be achieved either by applying high *A*_*c*_ for a short *t* or low *A*_*c*_ for a prolonged *t*. As reported by Paulsson and Ragauskas^23^ and Li et al.,^24–26^ appropriate alkaline pre-impregnation promotes lignin dissolution, whereas sufficient active alkali during pulping is crucial for sustaining delignification. At high *t* and *A*_*c*_, accelerated alkali consumption during prolonged pre-impregnation at high *A*_*c*_ may have reduced the effective alkali available during the subsequent soda-AQ pulping stage, thereby limiting overall lignin removal. Conversely, when both *t* and *A*_*c*_ were applied at lower levels, insufficient chemical penetration into the fiber structure may have contributed to the relatively higher kappa number of 13.2.

**Figure 1:**
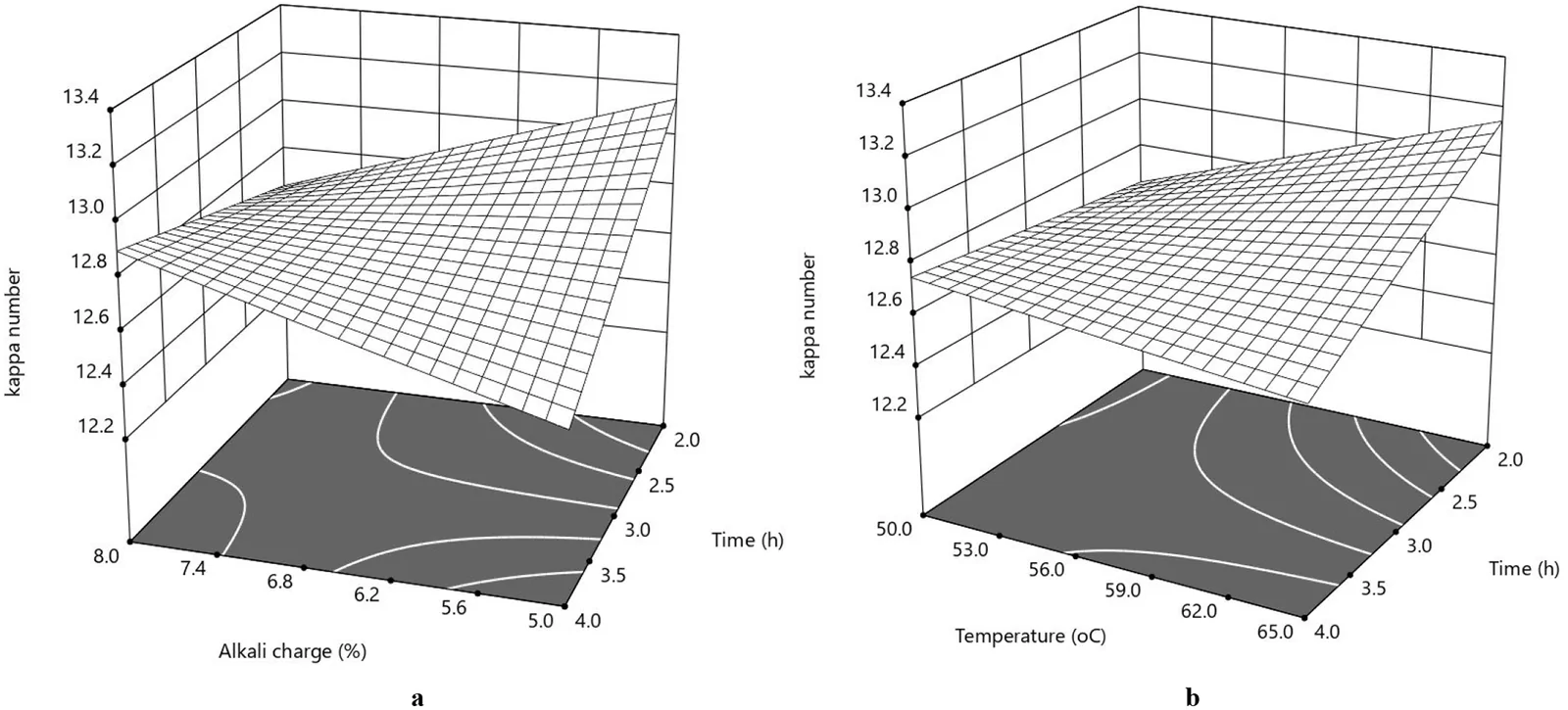
3D response surface plot for kappa number showing the interactive effects of **a** impregnation time (*t*) and alkali charge (*A_c_*) at a fixed temperature of 57.5 °C and **b** impregnation time (*t*) and temperature (*T*) at a fixed alkali charge (*A_c_*) of 6.5%.

The interactive effect of *Tt* was shown in Figure 1b. At 50.0 °C, a relatively low kappa number was obtained regardless of the impregnation time. In contrast, a high *T* (65.0 °C) for 2.0 h showed an adverse effect, resulting in the highest kappa number of 13.1. Under this condition, more alkali may have been consumed during impregnation than at 50.0 °C, resulting in a lower effective alkali during the pulping stage; consequently, less extensive delignification. Nevertheless, prolonging *t* to 4.0 h at 65.0 °C improved overall delignification to a similar level as in low *T*. The longer *t* may have promoted delignification more uniform alkali penetration and allowed delignification to commence during the impregnation stage. After the remaining alkali was added, delignification continued during the pulping stage, thereby improving the overall delignification efficiency.

#### 3.2.3 Pulp Viscosity

Pulp viscosity, commonly expressed in centipoise (cP), is widely used as an indirect indicator of the average molecular weight (*M*_w_) and degree of polymerization (DP) of cellulose chains in biomass, particularly in dissolving pulp with a low hemicellulose content. Therefore, a decrease in viscosity generally reflects cellulose degradation during a particular chemical treatment. However, the measured pulp viscosity may also be influenced by the presence of lower-molecular-weight hemicellulose, which generally have considerably much shorter chains than cellulose, with typical DP values ranging from approximately 50 to 200. Thus, a reduction of pulp viscosity after treatment may be associated with cellulose degradation, increased retention of low-DP hemicellulose, or a combination of both.

Compared to the one-stage soda-AQ pulp, all the two-stage soda-AQ pulps exhibited a decrease in pulp viscosity. Considering their higher pulp yield, the reduction in viscosity was most likely associated with the greater retention of low-DP hemicellulose.^29^ This is supported and verified by the increased preservation of hemicellulose content as shown in Table 5. During the first impregnation stage, part of the alkali has been consumed by various reactions, including limited polysaccharides peeling and competing stopping reactions. Consequently, the effective alkali concentration during the subsequent pulping stage may have been lower, while the prolonged impregnation allowed more gradual and uniform alkali penetration into the biomass than in the corresponding one-stage soda-AQ pulping. This moderated alkali exposure may have reduced hemicellulose peeling and dissolution at 160 °C, thereby contributing to greater hemicellulose retention in the two-stage pulps. In addition, some hemicellulose chains may have undergone reducing-end groups stabilization through stopping reactions, causing them less susceptible to further peeling during the subsequent pulping stage.

The factorial model (Table 3, eq 12) shows that all primary factors significantly influenced pulp viscosity (p < 0.05). The positive coefficients of temperature (*T*) and time (*t*) indicated that the increase in *T* and longer *t* resulted in higher pulp viscosity. Under the more severe impregnation conditions, greater fiber swelling, deacetylation and dissolution of accessible hemicellulose fraction may have reduced the amount of hemicellulose retained in the resulting pulp. As a result, the contribution of retained hemicellulose to the reduction in pulp viscosity became less pronounced, resulting in comparatively higher viscosity values. In contrast, alkali charge (*A*_*c*_) exhibited a negative effect on pulp viscosity, suggesting that an increase in *A*_*c*_ decreased the pulp viscosity. Among the interaction terms, only temperature–alkali charge (*TA*_*c*_) was statistically significant (p = 0.0202), whereas the time–temperature (*Tt*) interaction was not significant (p = 0.0609).

Interaction of *TA*_*c*_ exerted a positive effect on the pulp viscosity, as shown in Figure 2a. Most combinations of *T* and *A*_*c*_ resulted similar viscosity values of approximately 24.36–25.03 cP, which were only slightly lower than that of the one-stage soda-AQ pulp (26.16 cP). However, a distinct reduction of pulp viscosity to 22.45 cP was observed in the region combining a high *A*_*c*_ of 8.0% and low *T* of 50.0 °C, indicating that this impregnation condition produced the most pronounced decrease in pulp viscosity. As discussed earlier, the first-stage impregnation may have reduced the effective alkalinity during the subsequent pulping stage, thereby resulting in greater retention of hemicellulose than in the one-stage pulp.

**Figure 2:**
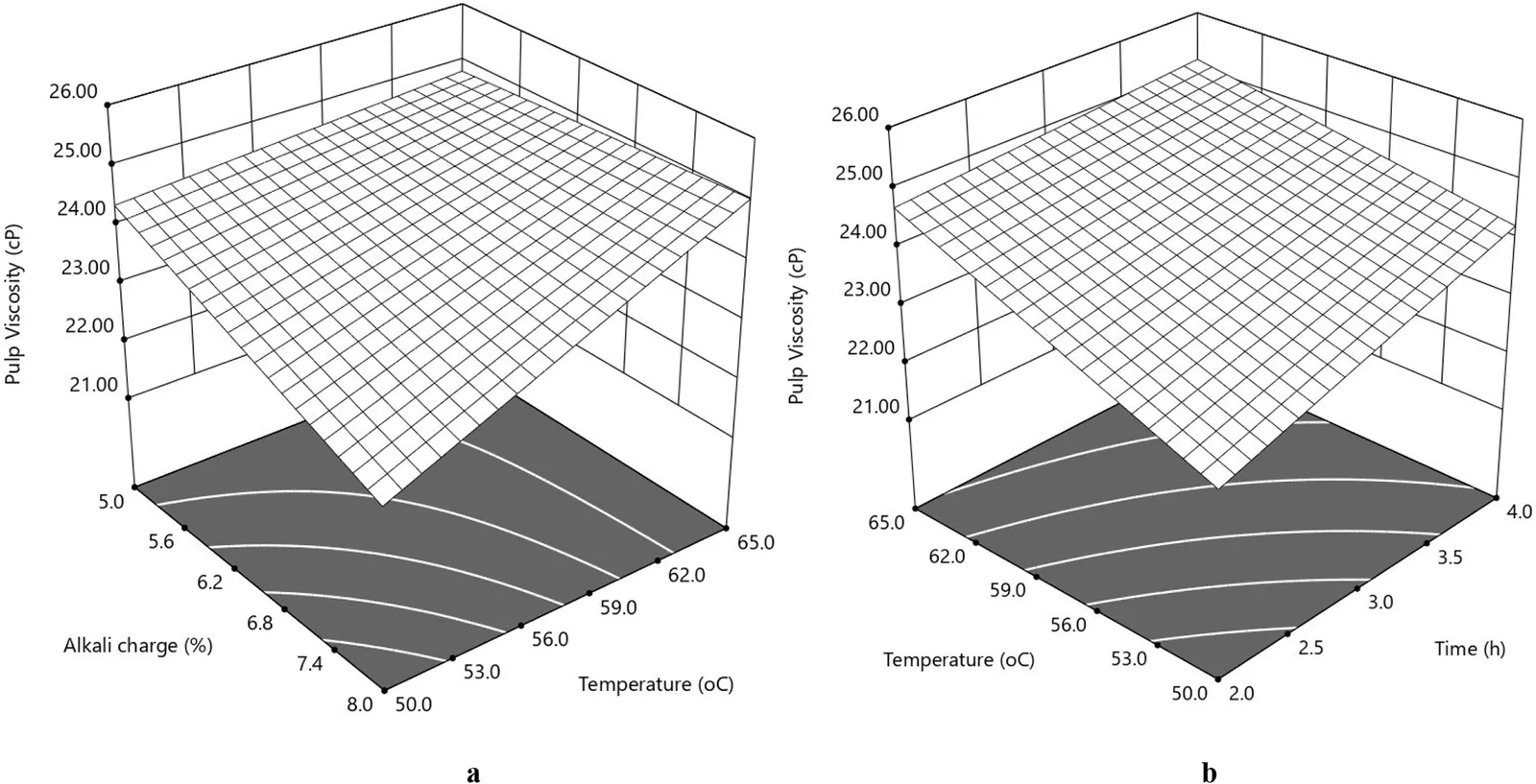
3D response surface plot for pulp viscosity showing the interactive effects of **a** impregnation temperature (*T*) and alkali charge (*A_c_*) at a fixed time of 3.0 h and **b** impregnation time (*t*) and temperature (*T*) at a fixed alkali charge (*A_c_*) of 6.5%.

This hemicellulose-retention effect, as reflected by the lower pulp viscosity, was most pronounced at 8.0% *A*_*c*_ and 50.0 °C, but least pronounced at 8.0% *A*_*c*_ and 65.0 °C. At the high-*A*_*c*_ and high-*T* region, greater fiber swelling, deacetylation and dissolution of the accessible hemicellulose fraction during impregnation may have reduced the amount of hemicellulose retained in the resulting pulp. The relatively higher viscosity in this region may also have been partly associated with reduced cellulose degradation, as Ang et al.^20^ reported that elevated pre-impregnation temperatures accelerated alkali consumption and reduced the effective alkalinity available during high-temperature pulping, thereby limiting cellulose degradation. In contrast, at 8.0% *A*_*c*_ and 50.0 °C, more alkali may have been consumed during the first stage, while the lower temperature provided insufficient energy for extensive hemicellulose dissolution. Consequently, more hemicellulose was retained in the pulp, resulting in the most pronounced reduction in pulp viscosity.

Although the *Tt* interaction was not statistically significant at the 5% level (*p* = 0.0609), it was retained in the model because its removal affected model stability and the estimated main-effect coefficients. As shown in Figure 2b, increasing impregnation time caused a similar magnitude of change in pulp viscosity at both *T* levels, while the effect of temperature was also comparable at both *t*. This near-parallel response indicates a weak *Tt* interaction, despite the significant individual effect of time. The lowest viscosity, approximately 22.45 cP, was predicted at the low *T* and short *t* region, which may be consistent with greater retention of hemicellulose. In contrast, the maximum viscosity of approximately 25.03 cP was obtained at 65.0 °C and 4.0 h, consistent with the positive coefficients of the individual T and t terms. The higher viscosity under this relatively more severe condition may have resulted partly from greater dissolution of the accessible hemicellulose fraction.

### 3.3 Effects of Pre-Impregnation Variables on Paper Properties

#### 3.3.1 Tensile Index

As referred to eq 13 (Table 3), only the alkali charge (*A*_*c*_) was statistically significant for tensile index (p = 0.0455), while temperature (*T*) and impregnation time (*t*) were retained in the model to maintain hierarchy despite being statistically insignificant. The lack of significance for *T* and *t* suggests that their linear effects alone exert only a minor influence on fiber flexibility and bonding. Meanwhile, the interaction terms temperature–time (*Tt*), temperature-alkali charge (*TA*_*c*_), and time-alkali charge (*tA*_*c*_) showed significant interactive effects on the tensile index, with only *tA*_*c*_ negatively influencing the tensile index.

Interaction *Tt* exerted a positive effect on the tensile index. As illustrated in Figure 3a, at a high *T* (65.0 °C), increasing *t* slightly increased the tensile index from 18.27 N m/g to 19.39 N m/g. In contrast, at low *T* (50.0 °C), extending *t* resulted in a pronounced decrease in tensile index from 19.00 N m/g to 16.96 N m/g. This indicates that the effects of pre-impregnation parameters on tensile index follow a trend similar to that observed for kappa number (see Figure 1b). Conditions that facilitate effective delignification (i.e., lower kappa number) enhanced the tensile strength by improving fiber flexibility and conformability.

**Figure 3:**
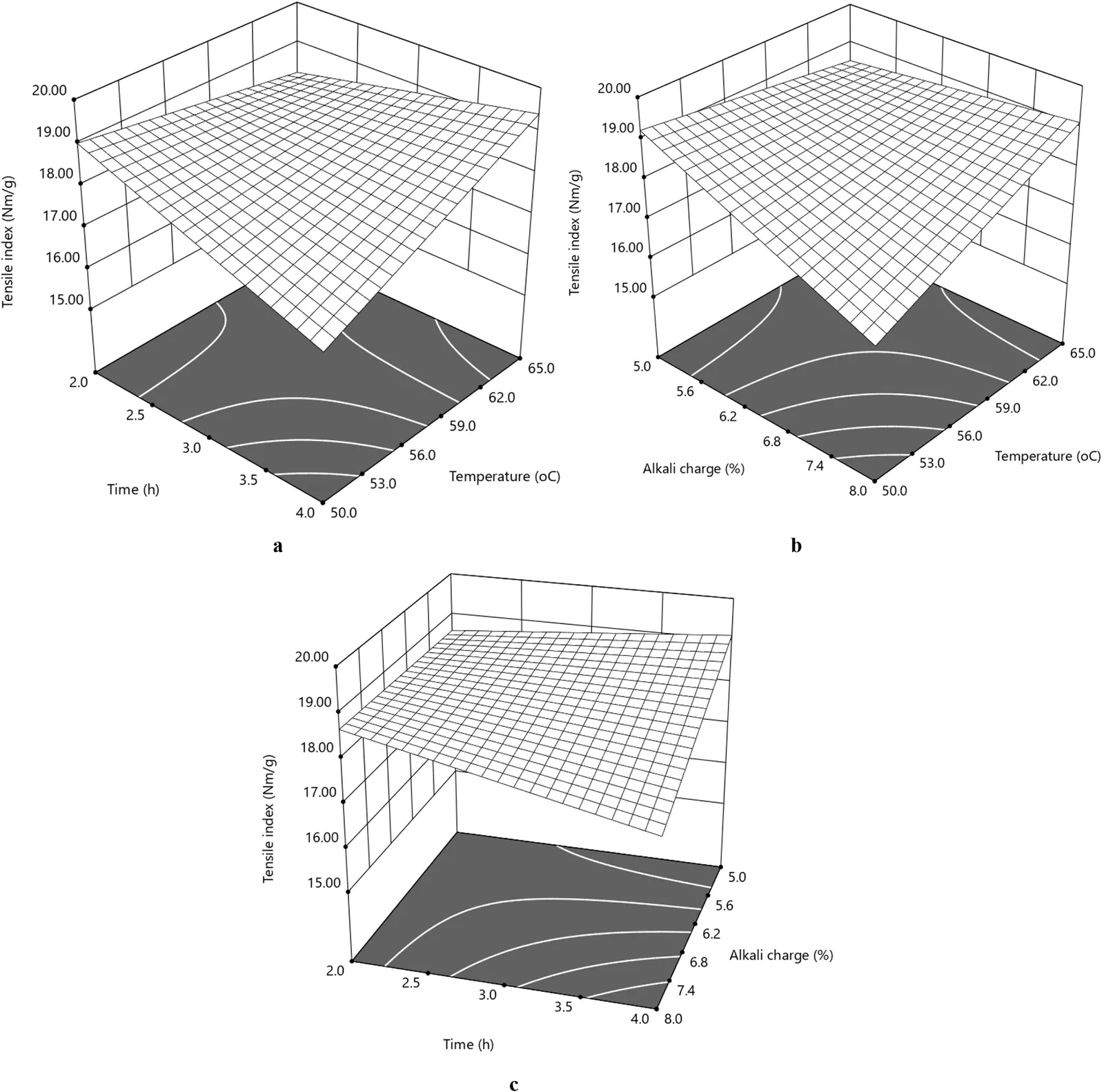
3D response surface plot for tensile index showing the interactive effects of **a** impregnation temperature (*T*) and time (*t*) at a fixed alkali charge (*A_c_*) of 6.5% and **b** impregnation temperature (*T*) and alkali charge (*A_c_*) at a fixed time (*t*) of 3.0 h and **c** alkali charge (*A_c_*) and impregnation time (*t*) at a fixed temperature (*T*) of 57.5 °C.

Figure 3b presents the 3D response surface plots of tensile index as a function of *T* and *A*_*c*_. At 65.0 °C, increasing *A*_*c*_ caused only a marginal increase in tensile index from 18.55 N m/g to 19.12 N m/g, whereas at 50.0 °C, increasing *A*_*c*_ led to a marked decrease in the tensile index from 19.21 N m/g to 16.77 N m/g. The lowest tensile index was observed at the high *A*_*c*_ with low *T*, where the lowest pulp viscosity (22.45 cP) was recorded under the same conditions as discussed earlier (see Figure 2a). This trend actually contradicts the general knowledge, in which increased hemicellulose content typically improves the strength properties of handsheets. This phenomenon can be explained and supported by the relatively high pulp freeness (662 mL) and lowest density (0.3487 g/cm^3^) as shown in Table 4, Table S1, Table S2, Figure S1 and Figure S2 for this particular condition, which suggested that the prolonged low-temperature impregnation caused chemical changes without sufficiently open up the fiber wall. Thus, during the subsequent soda-AQ pulping, the fibers remained intact and stiff, which suppressed fiber swelling and fibrillation required for fiber bonding. This porous network of fibers results in faster drainage and the formation of more bulky handsheets with weaker strength. Further study is required for evaluation.

**Table 4:** Effects of Alkaline Pre-Impregnation-Soda-AQ Pulping with and without Inter-Stage Washing on Pulp and Paper Properties

		APSP				APSPW			
		2.0 h		4.0 h		2.0 h		4.0 h	
	SP	5.0%	8.0%	5.0%	8.0%	5.0%	8.0%	5.0%	8.0%
Pulp Yield, %	50.0	52.5	52.5	51.9	53.8	52.9	52.5	52.3	56.2
Kkappa number	13.9 ± 0.1	12.9 ± 0.2	12.4 ± 0.1	12.5 ± 0.2	13.0 ± 0.2	13.1 ± 0.3	11.3 ± 0.2	10.4 ± 0.1	10.7 ± 0.2
Pulp viscosity, cP	26.16 ± 0.43	23.67 ± 0.75	21.44 ± 0.02	25.05 ± 0.16	23.42 ± 0.39	21.44 ± 0.86	21.71 ± 0.76	22.91 ± 0.23	22.41 ± 0.19
CSF, mL	569	633	626	640	662	626	608	603	606
Thickness, mm	0.1664 ± 0.0037	0.1584 ± 0.0054	0.1638 ± 0.0049	0.1585 ± 0.0043	0.1731 ± 0.0031	0.1643 ±0.0030	0.1510 ± 0.0024	0.1555 ± 0.0050	0.1599 ± 0.0043
Grammage, g/m^2^	60.65	59.75	60.18	58.64	60.37	58.77	58.86	59.65	59.83
Density, g/cm^3^	0.3645	0.3773	0.3673	0.3700	0.3487	0.3578	0.3897	0.3836	0.3742
Tensile Index, Nm/g	16.89 ± 0.24	19.79 ± 0.60	18.24 ± 0.41	18.64 ± 0.88	15.25 ± 0.84	16.74 ± 0.76	18.93 ± 0.13	21.57 ± 0.19	22.68 ± 0.97
Burst Index, kPa·m^2^/g	3.24 ± 0.33	3.52 ± 0.29	3.62 ± 0.29	3.55 ± 0.28	3.46 ± 0.28	3.72 ± 0.12	3.90 ± 0.17	4.21 ± 0.10	4.40 ± 0.27
Tearing Index, mN m^2^/g	5.95 ± 0.33	5.95 ± 0.34	6.20 ± 0.32	6.87 ± 0.38	6.90 ± 0.18	6.70 ± 0.29	8.43 ± 0.11	8.36 ± 0.63	7.39 ± 0.66
Brightness, %	42.43 ± 0.35	43.91 ± 0.31	46.16 ± 0.30	44.20 ± 0.26	43.59 ± 0.45	42.67 ± 0.34	42.74 ± 0.50	43.40 ± 0.34	44.79 ± 0.40
TAPPI Opacity, %	93.47 ± 0.89	93.77 ± 0.43	94.47 ± 0.65	94.58 ± 0.65	95.46 ± 0.73	96.72 ± 0.42	97.3 ± 0.76	96.85 ± 0.34	95.60 ± 0.46
Print Opacity, %	96.52 ± 0.51	96.61 ± 0.29	97.53 ± 0.58	97.71 ± 0.33	97.78 ± 0.66	98.46 ± 0.89	98.45 ± 0.66	97.90 ± 0.32	97.48 ± 0.36

Finally, the interaction trend between *t* and *A*_*c*_ exerted a negative effect on the tensile index. As illustrated in Figure 3c, at short *t* (2.0 h), the increase of *A*_*c*_ only resulted in a marginal increase of tensile index from 18.62 N m/g to 18.64 N m/g. On the other hand, at long *t*, the increase of *A*_*c*_ resulted in a significant decrease of tensile index from 19.13 N m/g to 17.23 N m/g. The highest tensile index (19.13 N m/g) achieved at low *A*_*c*_ and long *t* mapped the lowest kappa number (12.5) as discussed in Section 3.1.2. This result further confirmed that at low *A*_*c*_, an extended impregnation time, *t* allows for sufficient alkali penetration into the fiber core. This uniform chemical distribution promotes optimum delignification, which in turn enhances fiber flexibility and conformability, thereby promoting inter-fiber bonding and a higher tensile strength in the resulting paper network.

#### 3.3.2 Burst Index

As shown in Table 3 (eq 14), all linear terms—impregnation time (*t*), temperature (*T*), and alkali charge (*A*_*c*_) were statistically significant for burst index (p < 0.05). Among these, only *T* exhibited a positive coefficient, indicating that higher temperature enhances burst index, likely through improved fiber flexibility and conformability during handsheet formation. In contrast, *t* and *A*_*c*_ displayed negative coefficients, suggesting that higher values of these factors individually reduced the burst index. In addition, two interaction terms, *tA*_*c*_ and *TA*_*c*_, were statistically significant.

Kadar Mydin and Mazlana Main^27^ reported that tensile breaking strength and bursting strength are interdependent, establishing that changes in factors influencing tensile strength correspond directly to shifts in burst strength. As illustrated in Figure 4a, the lowest burst index recorded (3.57 kPa.m^2^/g) was consistent with the lowest tensile index (17.23 N m/g) in Figure 3c under same conditions: high *A*_*c*_ and long *t*. This suggests that the initial stage of alkaline over-exposure has been reached, in which most alkali was consumed by the pulp, leading to reduced effective alkalinity for delignification in the subsequent soda-AQ stage. Thus, an elevated in kappa number and stiffer fibers that drastically reduced both tensile and burst indices. As evidenced in Figure 1a, these conditions resulted in the second-highest kappa number rather than the absolute maximum, indicating that the excess alkali primarily induced carbohydrate degradation rather than further delignification.

**Figure 4:**
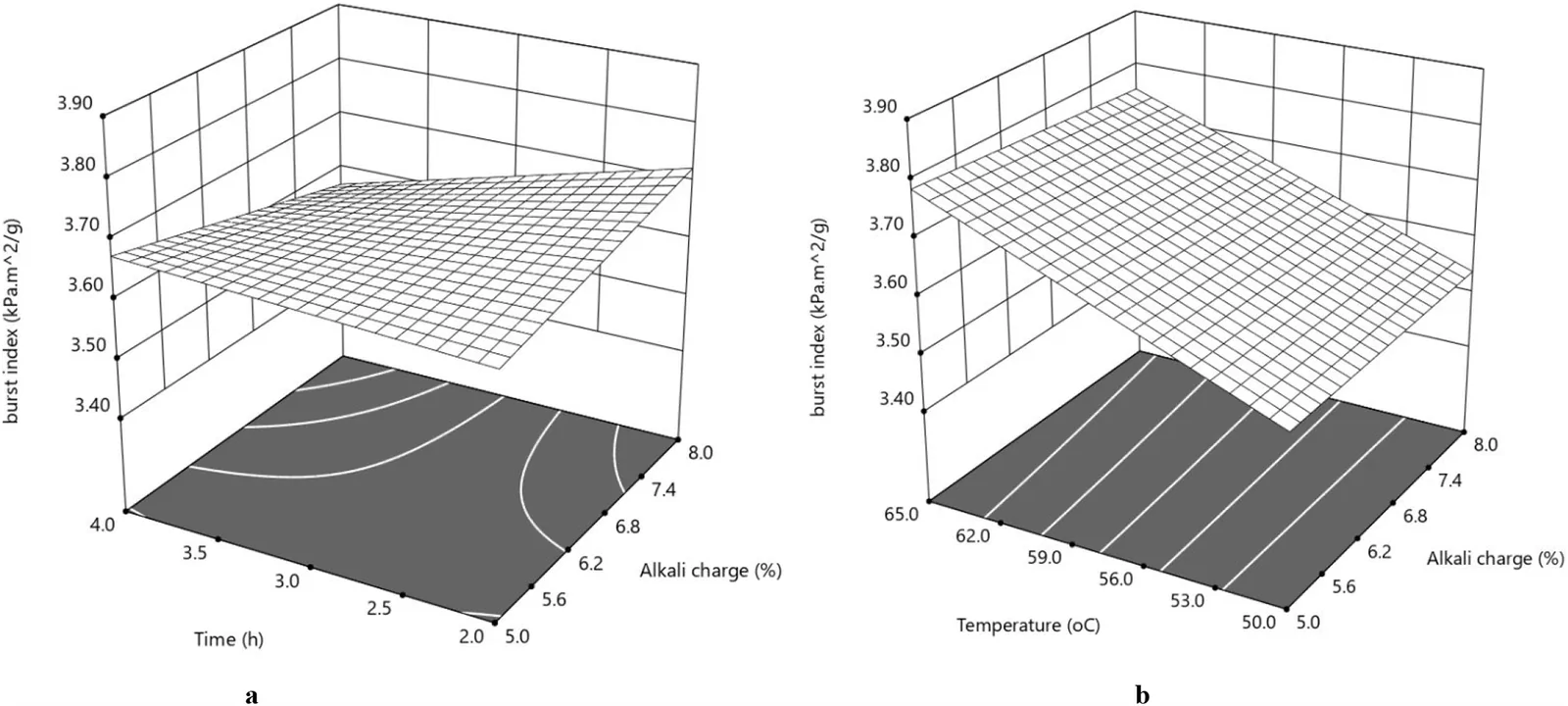
3D response surface plot for burst index showing the interactive effects of **a** alkali charge (*A_c_*) and impregnation time (*t*) at a fixed temperature (*T*) of 57.5 °C and **b** impregnation temperature (*T*) and alkali charge (*A_c_*) at a fixed time (*t*) of 3.0 h.

As shown in Figure 4b, another interaction term, *TA*_*c*_, exerted a negative effect on the burst index. At both low (5.0%) and high (8.0%) levels of *A*_*c*_, the burst index increased with *T*, which was consistent with the increase in pulp viscosity and tensile index under the same condition trends (see Figure 2a and Figure 4b). This suggested that *T* acted as a primary factor relative to *A*_*c*_ to accelerate the rate of alkaline consumption during the pre-impregnation stage, thus the residual alkalinity available for the subsequent soda-AQ pulping was reduced and severe cellulose depolymerization did not occur while retaining more hemicellulose content as verified in Table 5. However, as discussed earlier in Section 3.3.1, under this condition (high *A*_*c*_, low *T*) the pulp experienced chemical changes yet limited fibrillation. Therefore, the final handsheets were highly porous and bulky, with a little pressure the handsheet could burst.

**Table 5:** Gas Chromatography of SP, APSP, APSPW with All *T* Fixed at 50.0 °C and Alkaline Pre-Impregnation Conditions at 5A2H

Sugar	SP, %	APSP, %	APSPW, %
Arabinose	0.9	2.1	1.5
Xylose	22.3	21.3	22.8
Mannose	0.8	1.6	2.7
Galatose	0.9	1.1	0.5
Glucose	75.1	74.0	72.6

#### 3.3.3 Tearing Index

With reference to eq 15 and Table 3, all linear terms exhibited positive coefficients, indicating that increases in impregnation temperature (*T*), impregnation time (*t*), and alkali charge (*A*_*c*_) enhanced the tearing index. Among these, only *T* (p = 0.0075) and *t* (p = 0.0083) were statistically significant, whereas *A*_*c*_ showed the weakest influence (p = 0.1159). In addition to the linear effects, the temperature–time interaction (*Tt*) was statistically significant (p = 0.0476) with a negative coefficient (−0.1387).

As shown in Figure 5, increasing *T* enhanced the tearing index at both low and high levels of *t*, with a more pronounced increase observed at low *t*, as reflected by the steeper slope of the response surface. This behavior is consistent with the positive linear trends observed for pulp viscosity (see Figure 2b). Also, the highest tearing index recorded (7.16 mN·m^2^/g) aligns with the lowest kappa number (12.5) obtained under same conditions: 65.0 °C and 4.0 h as illustrated in Figure 1b. Thus, the tearing index is largely governed by the retention of cellulose degree of polymerization rather than delignification alone. Mechanistically, increasing *T* during the pre-impregnation stage accelerates alkali consumption, thereby reducing the effective alkali available for the subsequent soda-AQ pulping step. This moderates cellulose degradation and preserves polymer chain length, which contributes to improved tear resistance. At extended *t*, however, the available alkali becomes progressively depleted, which may promote carbohydrate peeling reactions and partial degradation of structural polysaccharides, thereby limiting further gains in tear strength.

**Figure 5:**
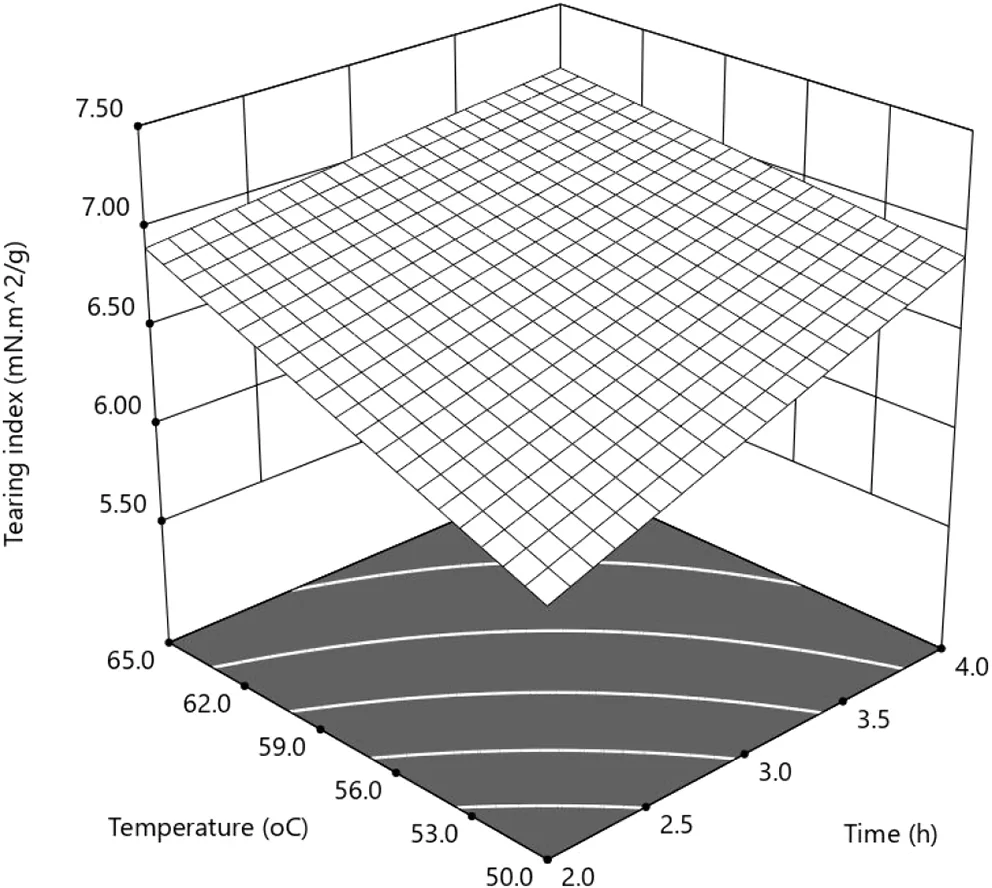
3D response surface plot for tearing index showing the interactive effects of impregnation temperature (*T*) and time (*t*) at a fixed alkali charge (*A_c_*) of 6.5%.

#### 3.3.4 Brightness

Based on eq 16 (Table 3), only alkali charge (*A*_*c*_) and impregnation time (*t*) are linear terms that are statistically significant with p = 0.0150 and 0.0209, respectively. *A*_*c*_ exerted a positive effect, whereas *t* exerted a negative effect on the brightness. Also, the time-alkali charge interaction (*tA*_*c*_) was statistically significant (p = 0.0213).

As shown in Figure 6, the interactive effect of *tA*_*c*_ was significant, where when *t* was at a high level, and *A*_*c*_ was at a low level, the increase in either *t* or *A*_*c*_ did not possess a noticeable effect on brightness. When *t* was long (4.0 h), alkaline darkening occurred on the biomass by generating chromophoric structure due to prolonged alkaline exposure limiting the brightness increment of the pulp produced.^28^ On the other hand, when the pre-impregnation was conducted for only 2.0 h, the increase of alkaline charge from 5.0% to 8.0% might improve the delignification. These results aligned with kappa number reduction, as shown in Figure 1a. In contrast, when considered individually, increasing *A*_*c*_ enhanced brightness, indicating more effective lignin removal and destruction of chromophoric groups responsible for pulp darkening.^29^ Thus, although higher alkali charge promotes brightness at moderate conditions, its combination with prolonged impregnation time reversed this effect due to alkaline darkening effect.

**Figure 6:**
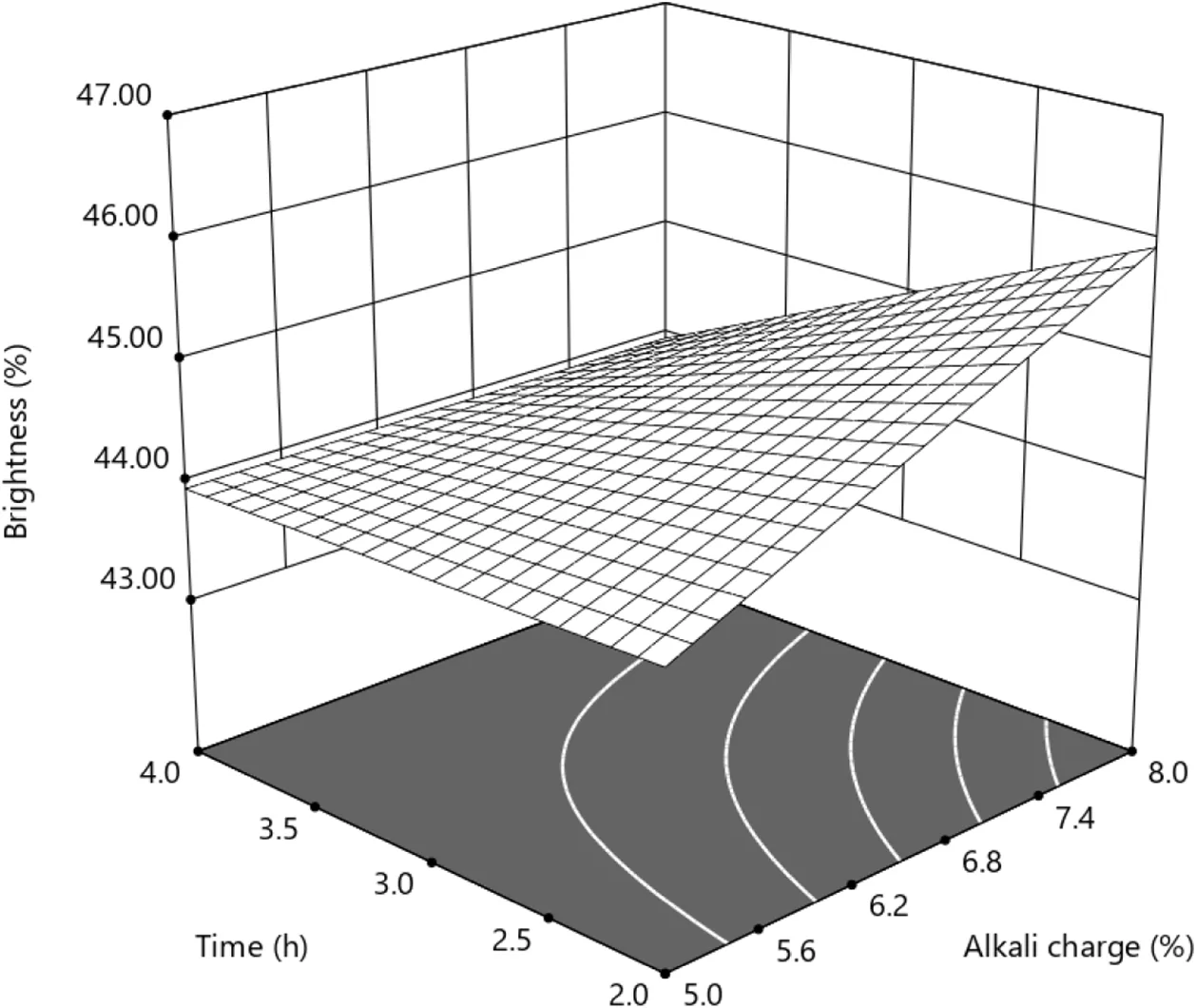
3D response surface plot for brightness showing the interactive effects of impregnation time (*t*) and alkali charge (*A_c_*) at a fixed temperature (*T*) of 57.5 °C.

### 3.4 Effect of Alkaline Pre-Impregnation-Soda-AQ Pulping with Inter-Stage Washing

Table 4 demonstrates the comparison between the one-stage soda-AQ pulping (SP), alkaline pre-impregnation – soda-AQ Pulping without inter-stage washing (APSP), and alkaline pre-impregnation – soda-AQ pulping with inter-stage washing (APSPW) on the pulp and paper properties. Crucially, although the 2^3^ factorial design showed that increasing temperature within the isolated pre-impregnation stage yielded positive effects, maintaining high thermal severity across a complete two-stage process risks over-processing the fibers during the final cook. Therefore, the pre-impregnation temperature was strategically fixed at a lower, less severe level (50.0 °C). This was intended to suppress carbohydrate degradation while the remaining conditions were anchored by the factorial model parameters. This minimized thermal energy consumption and provided a rigorous baseline proving that the subsequent performance surges were uniquely driven by the physical removal of dissolved organic debris rather than thermal intensity. Consequently, the washed sequence consistently outperformed both the SP and APSP, yielding a lower kappa number, lower freeness, higher density, and substantially improved tensile, burst, and tearing indices (Table 4).

These results indicate that inter-stage washing promoted more selective delignification while preserving structural carbohydrates. Based on Table 4, the simultaneous increase in pulp yield, reduced pulp viscosity and reduction of kappa number, suggests preferential lignin removal rather than cellulose degradation. For the increased pulp yield and reduced viscosity are directly supported by the carbohydrate composition verified in Table 5; specifically, the hemicellulose content (measured as xylose) increased progressively from SP (24.9%) to APSP (26.1%), and finally to the APSPW pulp (27.4%). Hemicellulose is readily solubilized under alkaline conditions and has a lower degree of polymerization compared to cellulose,^29^ and its retention contributes to fiber swelling and bonding.

This behavior is also attributed to the higher initial effective alkali concentration (HIEAC) provided by the introduction of fresh active alkali after the removal of spent liquor, replenishing the active chemical charge back to the required target level for the cooking stage. In contrast, in the unwashed sequence, a substantial fraction of the alkali charge was prematurely consumed via neutralization reactions with dissolved organic degradation products from the EFB fibers. According to Brännvall,^30,31^ higher HIEAC enhances hydroxide diffusion into the fiber matrix and ensures more uniform alkali distribution, preventing premature alkali depletion before reaching the fiber core. This promotes effective delignification while limiting cellulose peeling reactions. Furthermore, while the APSPW pulps exhibited a minor decrease in macromolecular pulp viscosity compared to their unwashed counterparts, this is attributed to a minor reduction in the cellulose degree of polymerization (DP) under high HIEAC,^1,3^ which was heavily counterbalanced by the extensive retention of hydrophilic hemicelluloses. The improved fiber swelling also explains the lower CSF values observed for the APSPW pulps.

Consequently, the improved pulp chemistry translated into superior paper properties. The washed pulps exhibited higher density, tensile, burst, and tearing indices. Effective delignification introduces carboxyl groups on fiber surfaces,^32,33^ enhancing electrostatic repulsion and fiber swelling, which promotes a highly conformable fiber network. Upon sheet formation, the retained amorphous hemicelluloses act as structural adhesives, strengthening inter-fiber adhesion due to their high surface area and hydrogen-bonding capability.^34,35^ This synergy explains the 34% to 40% improvements in tensile, burst, and tearing indices over the conventional SP.

Unexpectedly, brightness generally decreased after inter-stage washing, except under the most severe condition at 8% alkali charge and 4.0 h. Although alkali normally removes chromophoric lignin structures, new chromophores may form through lignin condensation and oxidation, absorbing blue light and darkening the pulp.^29^ Under extended reaction severity, these chromophores are further degraded, leading to the observed brightness recovery.

Regarding TAPPI opacity and print opacity of paper, both exhibited an increase after inter-stage washing, except under the most severe condition. This behavior is attributed to hemicellulose retention, which enhances fiber swelling and sheet densification, increasing light scattering and opacity.^36^ The opacity drop at severe conditions is consistent with brightness recovery due to extensive chromophore removal, which reduced light scattering through the internal pore spaces and air-fiber interfaces.

## 4 Conclusion

In this study, the 2^3^ factorial design successfully mapped and elucidated the complex multi-variable relationships governing the alkaline pre-impregnation of EFB prior to soda-AQ pulping. While total pulp yield was statistically invariant and independent of the factorial space, the remaining pulp and paper properties were systematically driven by critical main and two-way interaction effects. Temperature (*T*) was identified as the most critical determinant of pulp viscosity, burst, and tearing indices. Meanwhile, impregnation time (*t*) governed the kappa number and brightness, and alkali charge (*A*_*c*_) exerted the strongest influence on the tensile index. Beyond individual factors, two-way interaction dynamics heavily dictated process efficiency: *tA*_*c*_ synergy controlled the regulated the kappa number and sheet-bonding performance (tensile and burst indices) without sacrificing pulp yield. Concurrently, the *TA*_*c*_ interaction modulated macromolecular pulp viscosity and sheet cohesive strength, and the *Tt* combination broadly influenced delignification efficiency and fiber network properties. Crucially, for the tensile index, these multi-variable interactions exerted a more profound impact than the individual main effects of temperature and time, underscoring the highly synergistic nature of the pre-impregnation environment. By leveraging these factorial insights, a strategically designed sequence incorporating a low-temperature (50.0 °C) pre-impregnation step coupled with inter-stage washing was developed. This integrated framework achieved a superior balance between accelerated delignification and carbohydrate preservation and maintaining a high initial effective alkali concentration. This optimized process successfully yielded an exceptionally low kappa number of 10.4 alongside outstanding mechanical properties, including a tensile index of (22.68 N m/g), a burst index of (4.40 kPa·m^2^/g), and a tearing index of (8.36 mN·m^2^/g). Ultimately, this work provides a scalable, chemically efficient biorefinery pathway for the high-performance valorization of agricultural crop residues into robust bio-based materials.

## Supplementary Material



## Data Availability

The datasets generated during and/or analyzed during the current study are available from the corresponding author upon reasonable request.

## Notes

Table S1: density of handsheets for all 8 alkaline pre-impregnation conditions; Figure S1: effect of alkaline pre-impregnation variables on density of handsheets; Table S2: Canadian Standard Freeness (CSF) of handsheets for all 8 alkaline pre-impregnation conditions; Figure S2: effect of alkaline pre-impregnation variables on the Canadian Standard Freeness (CSF) of pulps (PDF)
