# Supplementary material for: Alkaline Pre-Impregnation and Inter-Stage Washing for Enhanced Soda-AQ Pulping of Oil Palm EFB
Source: ACS Omega. 2026 Sep 4;11(37):56001–13. doi: 10.1021/acsomega.6c05915 (PMC13613931; doi:10.1021/acsomega.6c05915)
Supplement: Supplementary file 1 [file ao6c05915_si_001.pdf]

Alkaline Pre-Impregnation and Inter-Stage Washing for Enhanced Soda-AQ Pulping of Oil Palm EFB

Yun Jie Chuah[1], Shi Teng Ng[1], Yitong Niu[1], Xiang Yi Liew[1], Cheu Peng Leh[1*]

[1]*Division of Bioresource Technology, School of Industrial Technology, Universiti Sains Malaysia, 11800 Minden, Penang, Malaysia*

***Corresponding Author:**
Cheu Peng Leh
Email: cpleh@usm.my

Supporting Information: Density and Canadian Standard Freeness (CSF) of the alkaline pre-impregnated soda-AQ pulps (two-stage soda-AQ pulping), including 8 conditions (5.0-8.0% alkali charge, 50.0-65.0 °C, 2.0-4.0 h) in the 2^3 Factorial Design in the Response Surface Methodology

Table S1: Density of handsheets for all 8 alkaline pre-impregnation conditions

	**Density, g/cm^3**			
	5.0 % alkali charge, 2.0 h	8.0% alkali charge, 2.0 h	5.0% alkali charge, 4.0 h	8.0% alkali charge, 4.0 h
control	0.3645			
50.0 °C	0.3773	0.3673	0.3700	0.3487
65.0 °C	0.3626	0.3676	0.3772	0.3701

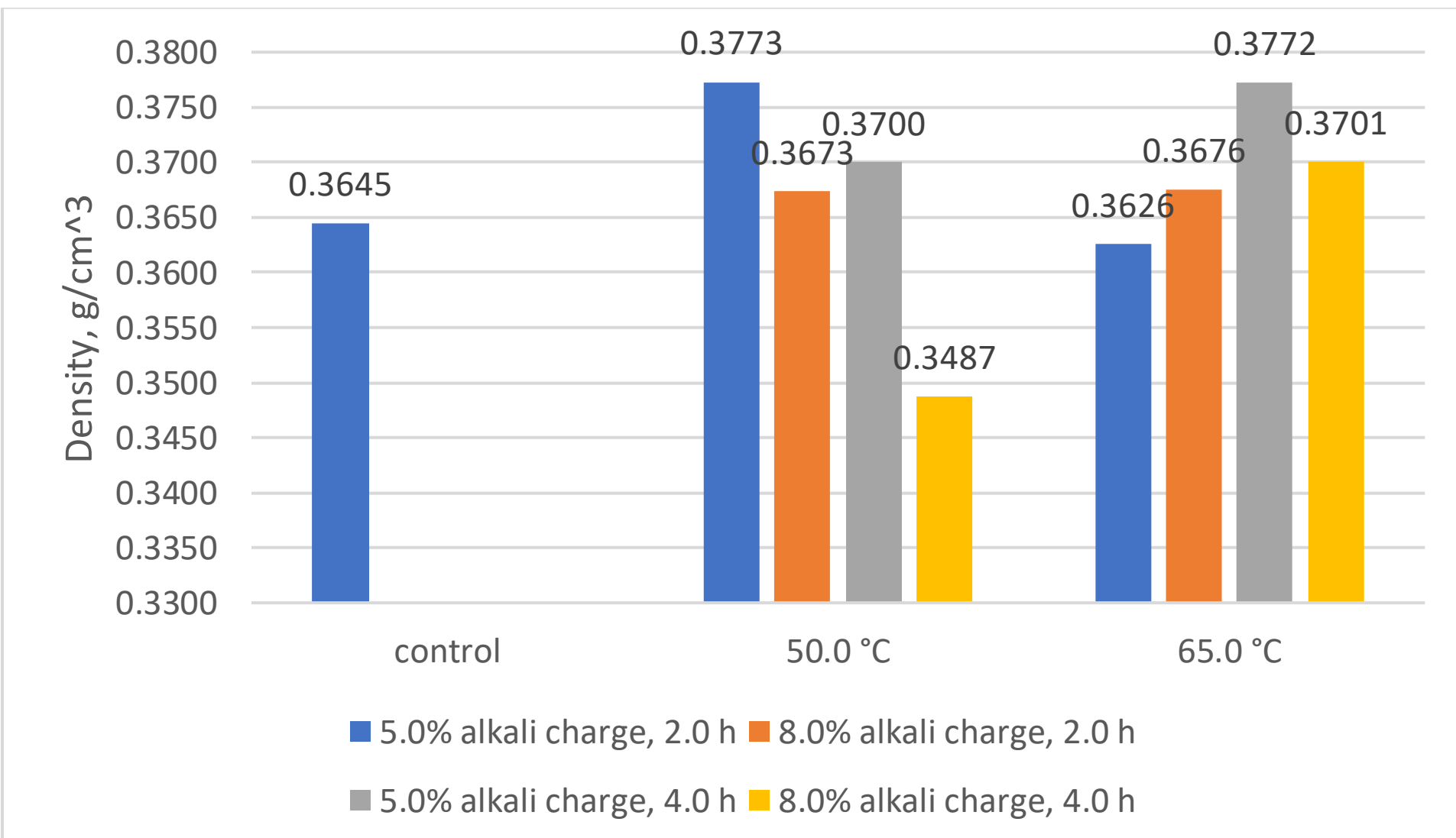

Fig. S1: Effect of alkaline pre-impregnation variables on density of handsheets

Table S2: Canadian Standard Freeness (CSF) of handsheets for all 8 alkaline pre-impregnation conditions

	**Canadian Standard Freeness (CSF), mL**			
	5.0% alkali charge, 2.0 h	8.0% alkali charge, 2.0 h	5.0% alkali charge, 4.0 h	8.0% alkali charge, 4.0 h
Untreated	569			
50.0 °C	633	626	640	662
65.0 °C	653	629	610	605

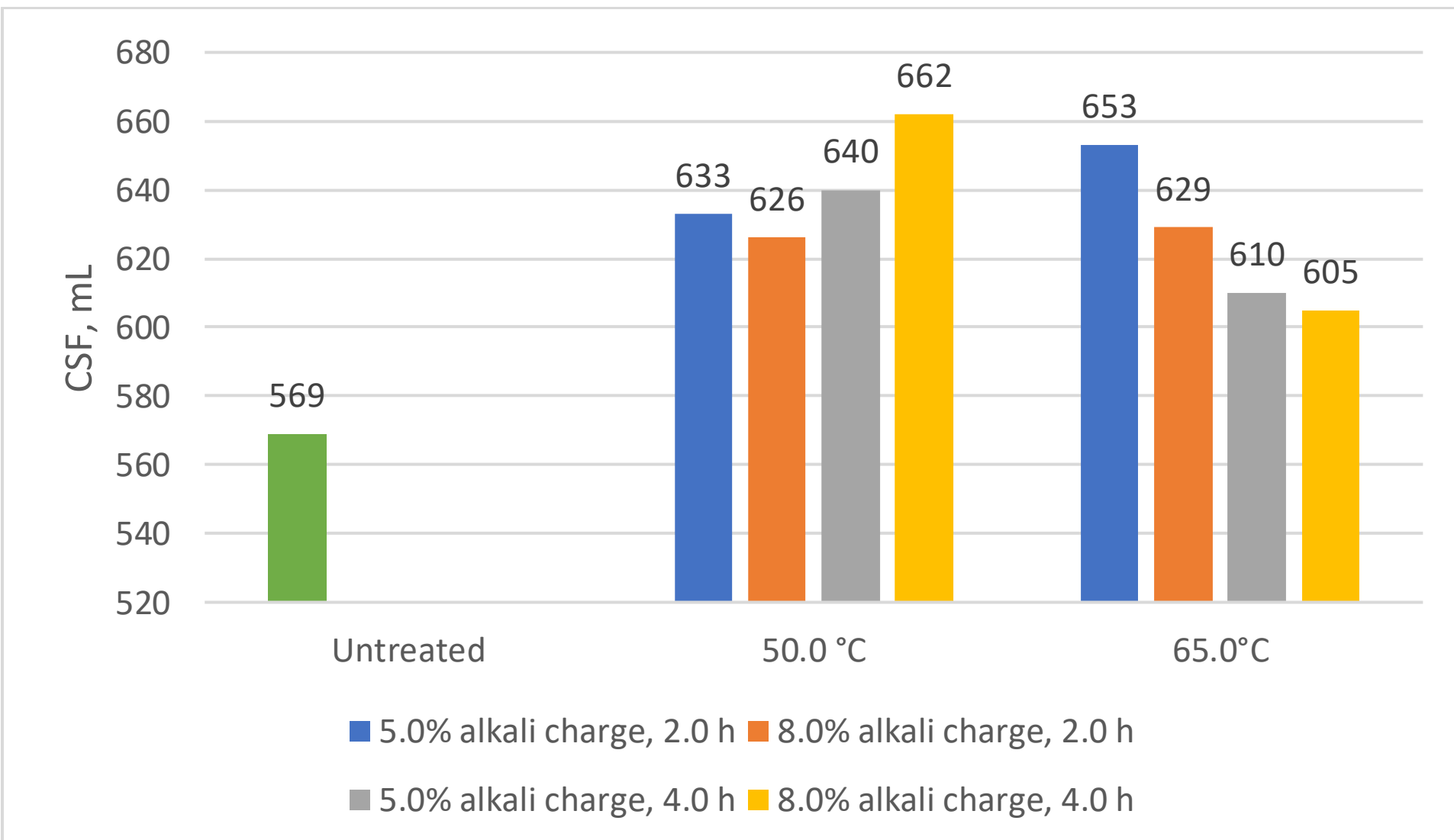

Fig. S2: Effect of alkaline pre-impregnation variables on the Canadian Standard Freeness (CSF) of pulps
